# Interpretable machine learning model for predicting covert hepatic encephalopathy in patients with cirrhosis: a multicenter study

**DOI:** 10.3389/fmed.2025.1686005

**Published:** 2025-11-25

**Authors:** Yilong Liu, Kai Ding, Yifan Qiu, Peiqin Wang, Ruoyao Wang, Xin Zeng, Chuan Yin

**Affiliations:** 1 Department of Gastroenterology, Changzheng Hospital, Naval Medical University, Shanghai, China; 2 College of Basic Medical Sciences, Naval Medical University, Shanghai, China; 3 Department of Gastroenterology, Shanghai East Hospital, Tongji University School of Medicine, Shanghai, China

**Keywords:** covert hepatic encephalopathy, machine learning, SHapley Additive exPlanations, cirrhosis, LightGBM

## Abstract

**Background and aim:**

Covert hepatic encephalopathy (CHE) is a neurocognitive complication affecting 40.9–50.4% of patients with cirrhosis. It often remains undiagnosed owing to its subclinical nature and the limitations of existing diagnostic tools, which are constrained by subjectivity, variable sensitivity, and limited accessibility. This study aims to develop and validate interpretable machine learning (ML) models for predicting CHE in patients with cirrhosis using multidimensional clinical and lifestyle data.

**Methods:**

This retrospective study included 503 patients with liver cirrhosis from 16 medical centers in China. CHE was diagnosed using the psychometric hepatic encephalopathy score and EncephalApp Stroop tests. Recursive feature elimination and Pearson’s correlation analysis were used for feature selection. Eight ML models were implemented to predict CHE. Performance was assessed via AUC, sensitivity, specificity, and decision curve analysis. The SHapley Additive exPlanations (SHAP) values are interpreted by the optimal model.

**Results:**

The light gradient boosting machine (LightGBM) model achieved the highest area under the receiver operating characteristic (ROC) curve (AUC) of 0.810 in the training set and 0.710 in the validation set. Decision curve analysis showed that LightGBM had better diagnostic performance than random forest (RF) and eXtreme gradient boosting (XGBoost). The SHAP analysis identified key predictors of CHE, including lower Mini-Mental State Examination (MMSE) scores, older age, hypoalbuminemia, lack of prior computer usage, and higher blood urea nitrogen levels.

**Conclusion:**

This study presents a novel ML-based approach for predicting CHE in cirrhotic patients, with LightGBM offering the best balance of performance and interpretability. The identified clinical and demographic predictors could facilitate early CHE detection and personalized management, ultimately improving outcomes for this high-risk population.

## Introduction

Covert hepatic encephalopathy (CHE), the subclinical precursor to overt hepatic encephalopathy (OHE), is a neurocognitive complication affecting 40.9–50.4% of patients with cirrhosis (1, 2). Characterized by subtle impairments in attention, visuospatial abilities, and psychomotor speed, CHE significantly compromises the quality of life and predicts hospitalization risks and mortality (3–6). In consequence, CHE often remains undiagnosed in routine clinical practice, which prevents the initiation of medical therapy. Early identification is critical for initiating interventions, such as lactulose or rifaximin, to mitigate progression (3, 7).

However, current diagnostic paradigms for CHE face substantial challenges. Owing to limited time, cost, and resource availability, only a few patients are routinely tested (8). Current diagnostic tools for CHE—including the psychometric hepatic encephalopathy score (PHES) and neuropsychological tests (NPTs)—are limited by subjectivity, variable sensitivity (PHES: 45–71%), and limited accessibility in routine clinical practice (9). Furthermore, these methods detect deficits only after neurological dysfunction is established, delaying therapeutic opportunities.

Blood biomarkers facilitating the diagnosis of covert hepatic encephalopathy (CHE) in patients with cirrhosis are lacking. While ammonia levels, inflammatory biomarkers (IL-6), and serum glial fibrillary acidic protein (sGFAP) have been investigated for minimal hepatic encephalopathy (MHE) prediction, their utility remains constrained by inconsistent thresholds and multifactorial pathophysiology (10–12). The heterogeneity of cirrhosis etiologies (e.g., viral, alcoholic, and metabolic dysfunction-associated steatotic liver disease) further complicates biomarker generalizability. Therefore, easy-to-use and reliable testing strategies are urgently required.

Machine learning (ML) offers transformative potential to decode complex patterns in multidimensional clinical data (13). By integrating neuropsychological parameters, serum biomarkers, and electronic health record trajectories, ML algorithms could help overcome the “silent” nature of CHE through predictive modeling. In recent years, some scholars have already utilized machine learning to predict or assist in the diagnosis of HE. Yang et al. demonstrated a weighted random forest (WRF) model achieving an AUC of 0.816–0.831 in predicting patients with liver cirrhosis complicated by HE (14). A recent study has demonstrated an ML model achieving an AUC of 0.825 in predicting post-transjugular intrahepatic portosystemic shunt (TIPS) overt hepatic encephalopathy (OHE) in patients with acute variceal bleeding (AVB), and the key predictors identified were Child–Pugh score, age, and portal vein thrombosis (15). However, at present, most of the studies on ML for predicting or diagnosing minimal hepatic encephalopathy focus on the use of imaging modules, while there is a lack of relevant research on predicting minimal hepatic encephalopathy using other clinical data (16, 17).

Therefore, this study aims to develop and validate interpretable ML models for CHE prediction in patients with cirrhosis, comparing their performance against conventional diagnostic tools, while identifying key predictive features across heterogeneous patient subgroups.

## Methods

### Study population

This is a retrospective study, with patient data derived from a multi-center cross-sectional study that enrolled 503 patients with liver cirrhosis at 16 medical centers in China between January 2021 and March 2022 (Supplementary Figure S1). Among the 503 patients with cirrhosis, 266 patients (52.88%) had covert hepatic encephalopathy (CHE) and 237 patients (47.12%) did not have CHE. To diagnose CHE, the standardized PHES, including five subtests, the app-dependent concise Color and Word Stroop tests, and the EncephalApp Stroop tests, were used (18, 19). PHES and the Stroop tests were performed according to the methods illustrated in previous studies (18, 19). The five subtests of PHES, number connection test A (NCT-A), number connection test B (NCT-B), line tracing test (LTT), serial dotting test (SDT), and digit symbol test (DST), were carried out by a trained investigator at each center. A total value of all subtests < 4 indicated a PHES positive result. The EncephalApp Stroop tests were administered with the same model of iPad in each center. The cutoff for the EncephalApp Stroop test was > 187 s for on time + off time (18, 19). When both the PHES and EncephalApp Stroop tests resulted positively, CHE was diagnosed. The study was performed in accordance with the Declaration of Helsinki (as revised in 2013). The study protocol was approved by the Institutional Ethics Committee of the Shanghai Changzheng Hospital (2020SL022). The protocol was explained to each patient, and informed consent forms were obtained from all individuals.

### Data collection and processing

The clinical and laboratory information of patients was retrieved from the medical records of participating hospitals (2). Features with over 25% missing values were excluded from the following analyses to minimize the bias resulting from missing data. Missing values were imputed using mean imputation, where the mean value of each respective feature was substituted for missing data points (20).

### Dataset preparation and feature selection

Prior to model development, continuous variables were standardized using z-score normalization based on the mean and standard deviation (SD) calculated from the training set. Categorical variables were binarized (1 indicating event presence and 0 representing absence), with gender specifically encoded as 1 for male and 0 for female. The complete dataset was partitioned into a training set (70%) for predictive model development and a test set (30%) for performance validation. The test set consisted of 84 patients with liver cirrhosis recruited from Changzheng Hospital from January 2024 to May 2025. To mitigate overfitting, 5-fold cross-validation was systematically implemented during model development. The recursive feature elimination (RFE) algorithm was used to select features from the data of the cohort. Pearson’s correlation coefficient was used to assess collinearity between variables.

### Model development and comparison

The features selected above were used to develop prediction models. Eight ML models, namely, adaptive boosting (AdaBoost), artificial neutral network (ANN), decision tree (DT), extra tree (ET), gradient boosting machine (GBM), light gradient boosting machine (LightGBM), random forest (RF), and eXtreme gradient boosting (XGBoost) were trained and established in the training cohort to predict CHE in liver cirrhosis.

Several commonly used evaluation indices, such as the area under the receiver operating characteristic (ROC) curve (AUC), sensitivity, specificity, positive predictive value (PPV), negative predictive value (NPV), accuracy, and F1 score, were used to evaluate the reliability of these models. The diagnostic performance of the model was evaluated using decision curve analysis (DCA).

### Model interpretation

The SHapley Additive exPlanations (SHAP) method was used to analyze the importance of features in the model because of the limited interpretability in the ML algorithm (21). SHAP was used as a scoring metric for feature contributions by determining the difference between the predicted values with and without each feature for all combinations. The greater the influence a particular value of a sample has on the composition of the model, the farther that point deviates from 0 on the x-axis. Using SHAP values and a summary plot, it is thus possible to determine which features have a significant effect on prediction and whether this contribution is positive or negative.

### Statistical analysis

Depending on the data distribution, the statistical significance of the difference in continuous variables was tested using Student’s *t*-test or Mann–Whitney U-test for quantitative variables and the chi-square test or Fisher’s test for qualitative variables. All statistical tests were two-sided, with *p*-values <0.05 indicating statistical significance. Statistical analyses and model development were performed using R software (version 4.05) and Python (version 3.8).

## Results

### Features selected from models

**Figure 1:**
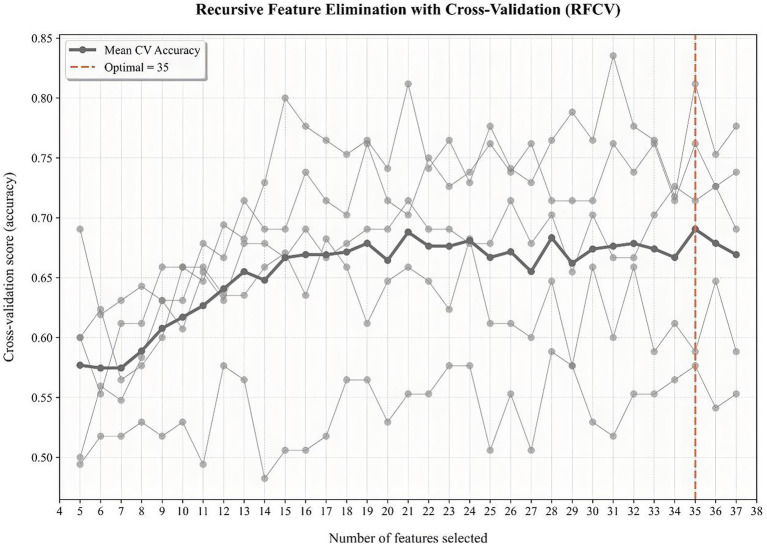
RFE coupled with 5-fold cross-validation to procure the most favorable combination of features.

To identify for the optimal subset to procure the most favorable combination of features, we used recursive feature elimination (RFE) coupled with 5-fold cross-validation. RFE enhances the performance of predictive models by eliminating overfitting and improving the generalizability of the model. According to a specific feature ranking standard, RFE starts from a complete set and then eliminates the least relevant feature one by one to select the most important features. Finally, 35 features achieved the highest cross-validation score, including age, education, sex, body mass index (BMI), history of OHE, etiology of liver disease, the course of liver cirrhosis, comorbidities of cardiovascular diseases, hypertension, cerebral apoplexy or diabetes, history of drinking, previous usage of smartphone, previous usage of computer, previous usage of tablet computer, medication history (L-ornithine aspartate, lactulose, probiotic formulations, non-selective *β* blockers, antiviral drugs, diuretic, rifaximin, metronidazole, and other antibiotics), alanine aminotransferase (ALT), aspartate aminotransferase(AST), alkaline phosphatase (AKP), g-glutamyltransferase (GGT), albumin (ALB), total bilirubin (TBil), creatinine (Cr), blood urea nitrogen (BUN), prolonged prothrombin time (PT), international normalized ratio (INR), and a series of health-related scores, namely, Mini-Mental State Examination (MMSE) score, Chronic Liver Disease Questionnaire (CLDQ) score, Child–Pugh score, and Model for end-stage liver disease (MELD) score (Figure 1).

### Pearson’s correlation of variables

**Figure 2:**
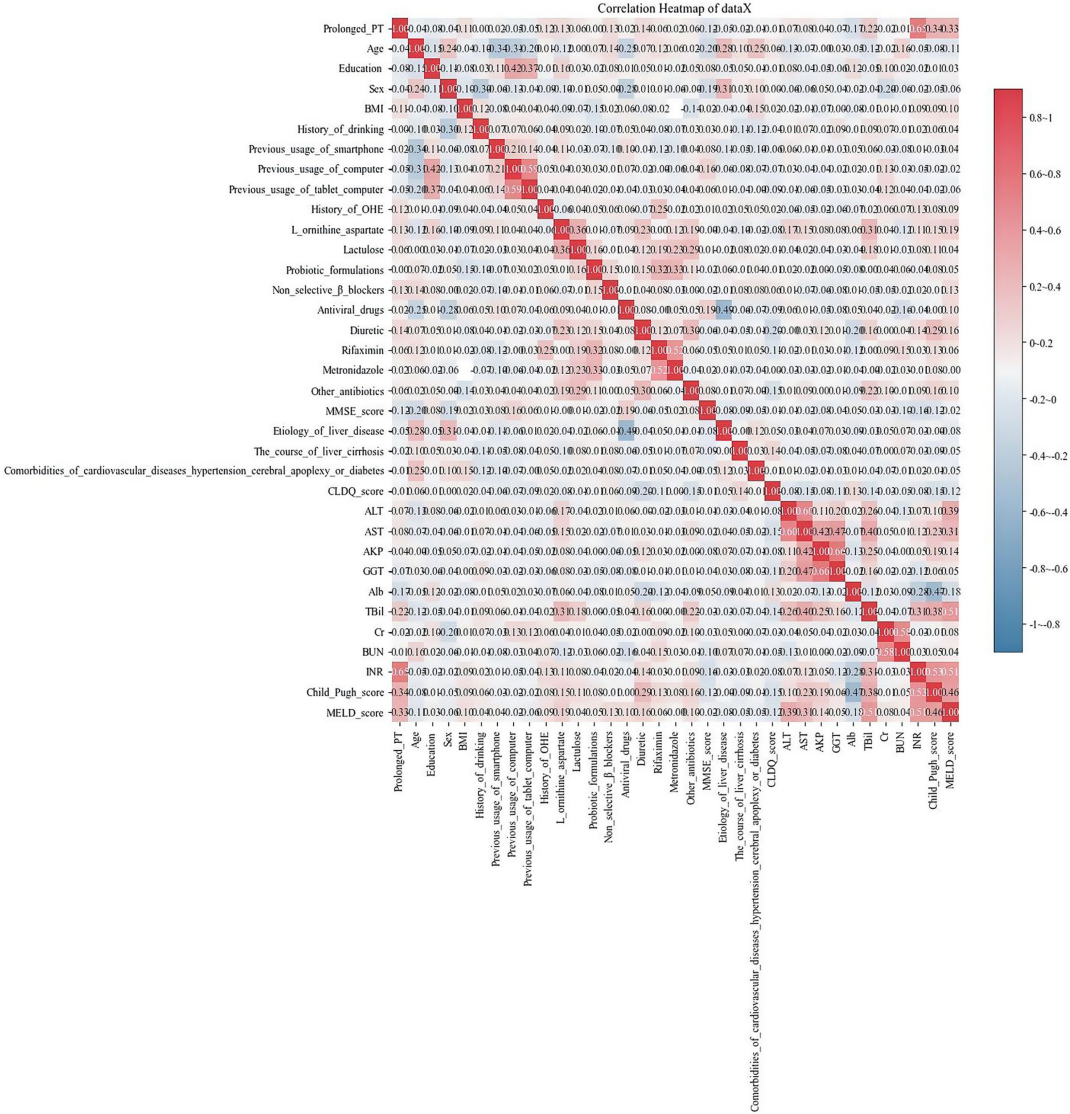
Heatmap shows the relationships among the variables. Each cell represents the correlation coefficient between two variables, ranging from −1 (perfect negative correlation, indicated by dark blue) to +1 (perfect positive correlation, indicated by dark red). A color bar on the right provides a reference scale.

We evaluated the correlations of variables using Pearson’s correlation and visualized the relationships among them through a heatmap (Figure 2). From the heatmap, we observed that there was collinearity between ALT and AST, AKP and GGT, and INR and prolonged PT. After discussion among gastroenterology experts, a decision was made to remove the three features of ALT, GGT, and INR.

### Patient characteristics

A total of 503 patients were allocated to separate training and validation sets at a ratio of 7:3. The demographic and clinical characteristics of the training and validation sets are listed in Table 1.

**Table 1:** Demographic and clinical characteristics of the training and validation sets.

Characteristics	Training set (*n* = 352)	Validation set (*n* = 151)	*p*
Age, years	51.29 ± 11.26	51.26 ± 11.47	0.946
Sex			
Male	260	117	0.432
Female	92	34	
BMI, kg/m^**2**^	23.62 ± 2.75	23.70 ± 2.60	0.894
Education, years	8.92 ± 3.19	9.12 ± 3.31	0.639
History of drinking			
N	253	101	0.287
Y	99	50	
Previous usage of a smartphone			
N	35	14	0.871
Y	317	137	
Previous usage of a computer			
N	247	114	0.236
Y	105	37	
Previous usage of a tablet computer			
N	289	133	0.112
Y	63	18	
Etiology of liver disease			
Hepatitis B virus	237	96	0.385
Hepatitis C virus	12	6	
Alcohol	33	13	
Primary Biliary Cholangitis	8	5	
Schistosoma	3	2	
Other	59	29	
The course of liver cirrhosis, day	900.72 ± 1661.32	764.34 ± 1208.55	0.534
Comorbidities of cardiovascular diseases, hypertension, cerebral apoplexy, or diabetes			
N	301	133	0.482
Y	51	18	
History of OHE			
N	344	147	0.758
Y	8	4	
CLDQ score	164.00 ± 26.22	165.19 ± 23.94	0.880
MMSE score	28.98 ± 1.20	29.03 ± 1.12	0.944
Child–Pugh score	7.25 ± 2.00	6.96 ± 1.82	0.172
MELD score	10.33 ± 6.59	10.32 ± 6.16	0.891
Medication history			
L ornithine aspartate			
N	259	110	0.913
Y	93	41	
Lactulose			
N	320	133	0.333
Y	32	18	
Probiotic formulations			
N	324	136	0.488
Y	28	15	
Non-selective β-blockers			
N	332	138	0.241
Y	20	13	
Antiviral drugs			
N	157	63	0.558
Y	195	88	
Diuretic			
N	251	114	0.383
Y	101	37	
Rifaximin			
N	338	147	0.604
Y	14	4	
Metronidazole			
N	348	150	>0.999
Y	4	1	
Other antibiotics			
N	312	131	0.551
Y	40	20	
Blood test			
AST, U/L	80.75 ± 119.53	78.22 ± 112.38	0.388
AKP, U/L	135.07 ± 95.08	120.52 ± 85.03	0.062
TBil, μmol/L	45.52 ± 67.98	46.25 ± 69.06	0.675
Alb, g/L	34.60 ± 7.00	35.02 ± 7.64	0.418
Prolonged PT, s	3.75 ± 7.62	3.14 ± 4.05	0.397
Cr, μmol/L	68.82 ± 26.68	70.60 ± 27.76	0.262
BUN, mmol/L	5.44 ± 2.92	5.53 ± 2.98	0.407
CHE			
N	171	66	0.331
Y	181	85	

Y, yes; N, no; BMI, body mass index; ALT, alanine aminotransferase; AST, aspartate aminotransferase; AKP, alkaline phosphatase; GGT, g-glutamyltransferase; ALB, albumin; TBil, total bilirubin; Cr, creatinine; BUN, blood urea nitrogen; PT, prothrombin time; INR, international normalized ratio; MMSE, Mini-Mental State Examination; CLDQ, Chronic Liver Disease Questionnaire; MELD, model for end-stage liver disease; CHE, covert hepatic encephalopathy; OHE, overt hepatic encephalopathy.

### Model development and validation

**Figure 3:**
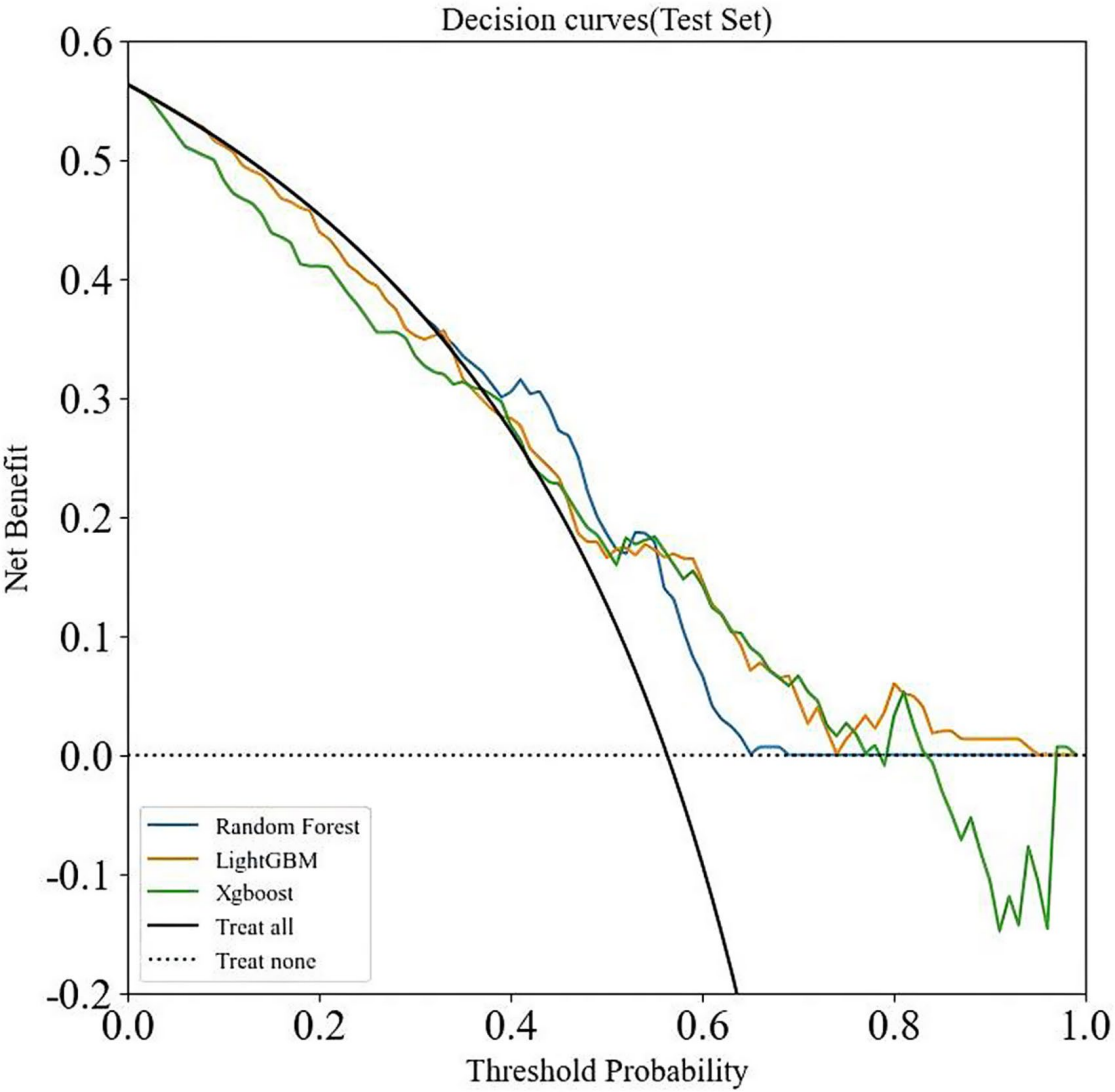
DCA analysis of three machine learning algorithms (LightGBM, RF, and XGBoost) in the validation set.

Eight ML models—AdaBoost, ANN, DT, ET, GBM, LightGBM, RF, and XGBoost—were constructed based on 32 features. As shown in Table 2, among these models, LightGBM, RF, and XGBoost exhibited higher AUC values (0.810, 0.797, and 0.801) compared to the others. These three models were further evaluated, with the results presented in Table 3. Supplementary Figure S2 shows the confusion matrices of the validation set. Additionally, an extensive decision curve analysis (DCA) demonstrated that LightGBM exhibited better diagnostic performance than RF and XGBoost in the test set (Figure 3). Given that LightGBM has the highest AUC value in the training set and performs well in the test DCA, we chose LightGBM as the final model for this study. In the independent test set, the LightGBM model achieved an outstanding AUC of 0.855 (0.852, 0.857), further confirming its robust generalization capability (Supplementary Table S1).

**Table 2:** Performance of the eight ML models in the training set.

Model	AUC	Sensitivity	Specificity	PPV	NPV	Accuracy	F1 Score
LightGBM	0.810 (0.779, 0.841)	0.694 (0.505, 0.883)	0.836 (0.629, 1.043)	0.852 (0.72, 0.983)	0.719 (0.588, 0.85)	0.758 (0.725, 0.792)	0.748 (0.683, 0.814)
RF	0.797 (0.758, 0.835)	0.746 (0.585, 0.907)	0.740 (0.592, 0.889)	0.773 (0.655, 0.892)	0.730 (0.602, 0.858)	0.742 (0.705, 0.779)	0.750 (0.675, 0.825)
XGBoost	0.801 (0.765, 0.838)	0.755 (0.595, 0.914)	0.78 (0.601, 0.959)	0.804 (0.673, 0.935)	0.751 (0.591, 0.911)	0.761 (0.731, 0.792)	0.768 (0.714, 0.822)
AdaBoost	0.774 (0.74, 0.808)	0.695 (0.497, 0.894)	0.72 (0.515, 0.925)	0.773 (0.671, 0.874)	0.689 (0.585, 0.793)	0.716 (0.69, 0.742)	0.716 (0.636, 0.795)
ANN	0.653 (0.582, 0.724)	0.624 (0.381, 0.867)	0.703 (0.494, 0.913)	0.731 (0.648, 0.815)	0.636 (0.591, 0.681)	0.676 (0.643, 0.709)	0.656 (0.503, 0.809)
DT	0.655 (0.575, 0.736)	0.659 (0.495, 0.823)	0.675 (0.493, 0.857)	0.707 (0.543, 0.871)	0.635 (0.506, 0.763)	0.656 (0.596, 0.717)	0.667 (0.588, 0.746)
ET	0.791 (0.743, 0.838)	0.82 (0.737, 0.902)	0.716 (0.574, 0.858)	0.772 (0.666, 0.877)	0.772 (0.671, 0.872)	0.767 (0.739, 0.795)	0.789 (0.767, 0.811)
GBM	0.703 (0.62, 0.786)	0.752 (0.575, 0.929)	0.634 (0.476, 0.793)	0.705 (0.564, 0.845)	0.708 (0.547, 0.869)	0.693 (0.618, 0.769)	0.718 (0.616, 0.819)

**Table 3:** Performance of the three ML models in the validation set.

Model	AUC	Sensitivity	Specificity	PPV	NPV	Accuracy	F1 Score
LightGBM	0.710 (0.708, 0.713)	0.748 (0.739, 0.757)	0.631 (0.622, 0.639)	0.689 (0.685, 0.694)	0.725 (0.72, 0.73)	0.69 (0.688, 0.692)	0.703 (0.7, 0.707)
RF	0.712 (0.71, 0.715)	0.851 (0.846, 0.855)	0.551 (0.546, 0.556)	0.665 (0.662, 0.668)	0.787 (0.783, 0.792)	0.704 (0.702, 0.706)	0.743 (0.741, 0.746)
XGBoost	0.711 (0.708, 0.713)	0.783 (0.778, 0.788)	0.612 (0.606, 0.618)	0.681 (0.677, 0.684)	0.737 (0.733, 0.742)	0.699 (0.697, 0.701)	0.723 (0.721, 0.726)

### Model interpretation

**Figure 4:**
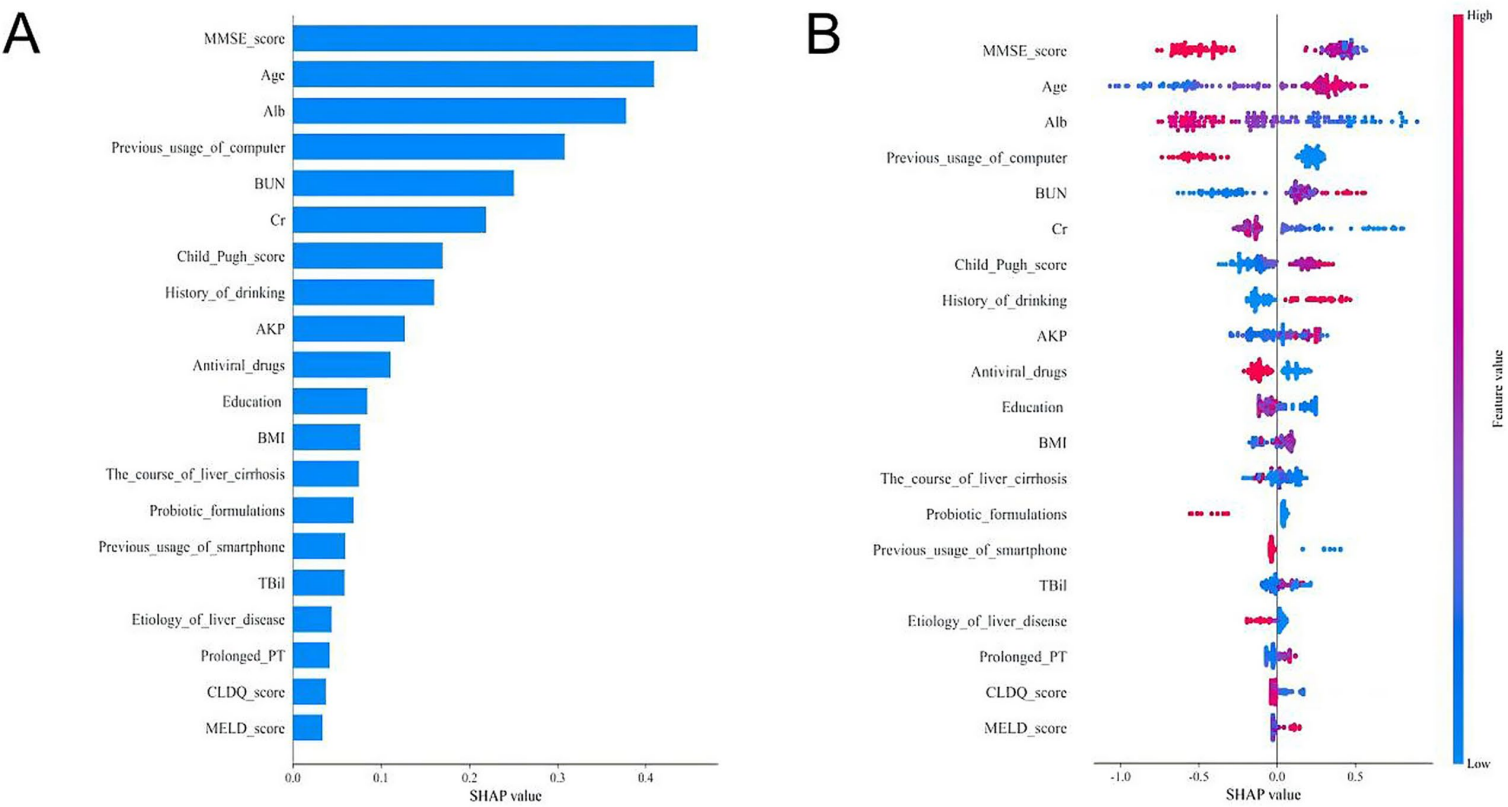
SHAP analyses of the LightGBM model for predicting CHE in cirrhotic patients. **(A)** Mean absolute SHAP value bar plot. This plot ranks the top 20 features by their mean absolute SHAP value, which represents their overall importance in the model’s predictions. A larger bar indicates a greater average impact on the model output. **(B)** SHAP summary plot (beeswarm plot). This plot shows the distribution of the impact each feature has on the model output for every patient in the dataset. Each dot represents a single patient.

To enhance the clinical utility of the model, we used the SHAP method to identify the features contributing to the prediction of CHE in patients with cirrhosis, as illustrated in Figure 4. The bar plot was generated by ranking features according to their mean absolute SHAP values in descending order, which reflects the relative contribution of each feature to the overall model. A higher absolute SHAP value indicates greater feature importance and a stronger influence on the model’s output.

As shown in Figure 4A, the top five clinically significant features were identified as MMSE score, age, ALB, age, previous computer usage, and BUN. Furthermore, we present the distribution of SHAP values for the top 20 clinical features, where each dot represents a feature (Figure 4B). The position of the dot indicates the SHAP value, quantifying the feature’s contribution to the model output. A positive SHAP value suggests a positive influence on the output, whereas a negative value indicates an inhibitory effect. Color intensity reflects feature magnitude—red denotes high values, while blue represents low values, with darker shades signifying a stronger impact on the target outcome.

It demonstrates that low MMSE scores, older age, low albumin levels, high blood urea nitrogen, and lack of prior computer usage were associated with an increased likelihood of CHE development (Figure 4B). This interpretability analysis enhances the model’s clinical applicability by identifying key predictive factors and their directional influence on CHE risk.

## Discussion

The clinical implications of CHE in patients with cirrhosis are profound, as it is associated with cognitive impairment, reduced quality of life, and an increased risk of OHE and mortality (1, 3). Early identification of CHE is crucial for timely intervention, yet its diagnosis remains challenging due to the lack of overt symptoms and the reliance on specialized neuropsychological tests such as the Psychometric Hepatic Encephalopathy Score (PHES) and EncephalApp Stroop tests (18, 22). In this study, we developed and validated multiple ML models to predict CHE in cirrhotic patients, leveraging a multicenter cohort of 503 cirrhotic patients.

To our knowledge, this is the first multi-center study to systematically evaluate eight ML models for predicting CHE in patients with cirrhosis using multidimensional clinical and lifestyle data. Our findings demonstrate that the LightGBM model outperformed other ML algorithms, achieving an AUC of 0.810 (0.779, 0.841) in the training set and 0.710 (0.708, 0.713) in the validation set, highlighting its potential as a reliable predictive tool for CHE. Notably, our model does not achieve comparable accuracy to advanced biomarker-driven approaches (e.g., MRI-based models), while relying solely on low-cost, less time-consuming parameters—a critical advantage in resource-limited settings (17, 23).

The SHAP-based interpretability framework revealed key predictors of CHE, including lower MMSE scores, older age, hypoalbuminemia, lack of prior computer usage, and higher levels of blood urea nitrogen. The prominence of MMSE scores underscores the need for early cognitive screening in cirrhosis management, while the association between hypoalbuminemia and CHE risk corroborates its role in hepatic synthetic dysfunction and neurotoxin accumulation (24). Previous studies have shown that older age is an independent biomarker associated with CHE (2). Older age may lead to increased defects in some areas of the central nervous system, affecting cognitive function and the development of CHE (25). This is also consistent with the results of a recent meta-analysis (1). Blood urea nitrogen can reflect renal function, and high levels of urea nitrogen are usually associated with poor protein catabolism, dehydration, and gastrointestinal bleeding. Some studies have shown that high urea nitrogen is an independent biomarker related to the severity and prognosis of HE (26, 27). An intriguing finding from our SHAP analysis was the importance of “previous computer usage” as a protective factor. We hypothesize that this variable acts as a practical proxy for cognitive reserve—the brain’s resilience to pathology. Engaging with computers is a complex cognitive activity that may help build neural networks that are more resistant to the neurotoxic insults of cirrhosis. While this factor likely correlates with socioeconomic status and education level—the latter of which was included in our model and provided independent information—its retention as a key predictor suggests that it captures a unique dimension of a patient’s cognitive lifestyle. This finding aligns with studies linking technology use to cognitive function in older adults (28). Although direct socioeconomic data were not available, this finding highlights the potential value of incorporating simple assessments of life engagement into risk stratification. Future prospective studies should aim to collect more detailed socioeconomic and lifestyle data to disentangle these complex relationships.

The clinical relevance of our model lies in its potential to streamline CHE diagnosis by integrating readily available clinical and laboratory variables, thereby reducing reliance on time-consuming neuropsychological tests. For instance, hypoalbuminemia, a key predictor in our model, is a well-established marker of liver synthetic dysfunction and is associated with blood–brain barrier disruption, facilitating neurotoxin accumulation (29, 30). Similarly, the MMSE score can, to some extent, reflect the interplay between global cognitive function and CHE (24).

Our study addressed critical gaps in CHE prediction research. First, the integration of lifestyle factors (e.g., digital device usage) with traditional clinical variables (e.g., MELD score) provides a holistic risk profile, capturing both biological and psychosocial determinants of CHE—a paradigm shift from prior biomarker-centric models. Second, the rigorous feature selection pipeline (RFE + expert-guided collinearity reduction) optimized model parsimony. For instance, excluding redundant variables (e.g., ALT and INR) improved generalizability without sacrificing predictive power.

Despite its strengths, our study has limitations. First, the retrospective design may introduce selection bias, and the reliance on imputation for missing data (though limited to variables with <25% missingness) could affect model generalizability. Second, while our retrospective design mitigated recall bias, the cohort was restricted to Chinese patients, necessitating external validation in global populations to confirm generalizability. Third, while the LightGBM model demonstrated good discrimination, its moderate AUC (0.710 in the validation set) suggests room for improvement, potentially through the inclusion of additional biomarkers such as gut microbiota profiles, inflammatory markers, or imaging parameters. Besides, the operational definition of “previous computer usage” requires standardization across diverse socioeconomic contexts. Future prospective studies should also explore the integration of dynamic variables (e.g., longitudinal cognitive assessments) to enhance predictive accuracy.

Despite these limitations, our LightGBM model offers a pragmatic tool for CHE risk stratification in routine practice. By prioritizing easily accessible variables (e.g., albumin and MMSE scores), it could be seamlessly embedded into the Hospital Information System (HIS) to guide targeted monitoring. For clinicians, the SHAP dashboard provides actionable insights, transforming opaque algorithms into transparent decision aids (31). Furthermore, we could develop an interactive SHAP visualization tool to translate model outputs into clinician-friendly risk assessments, bridging the “black-box” gap in ML applications. Such tools could be integrated into the HIS to trigger real-time alerts for high-risk patients.

In conclusion, our study presents a novel ML-based approach to CHE prediction, with the LightGBM model offering the best balance of performance and interpretability. By identifying key clinical and demographic predictors, this tool could facilitate early CHE detection and personalized management in cirrhotic patients. Future efforts should focus on external validation and the development of user-friendly applications to translate this model into clinical practice, ultimately improving outcomes for this high-risk population.

## Data Availability

The raw data supporting the conclusions of this article will be made available by the authors, without undue reservation.
