# Supplementary material for: Interpretable machine learning model for predicting covert hepatic encephalopathy in patients with cirrhosis: a multicenter study
Source: Front Med (Lausanne). 2025 Nov 25;12:1686005. doi: 10.3389/fmed.2025.1686005 (PMC12685911; doi:10.3389/fmed.2025.1686005)
Supplement: Supplementary file 1 [file Data_Sheet_1.docx]

**Supplementary Table 1.** Performance of the LightGBM in the Test set.

|  | **AUC** | **Sensitivity** | **Specificity** | **PPV** | **NPV** | **Accuracy** | **F1 Score** |
| --- | --- | --- | --- | --- | --- | --- | --- |
| **LightGBM** | 0.855 (0.852, 0.857) | 0.883 (0.879, 0.888) | 0.773 (0.768, 0.778) | 0.836 (0.833, 0.840) | 0.844 (0.839, 0.849) | 0.835 (0.833, 0.838) | 0.856 (0.853, 0.858) |


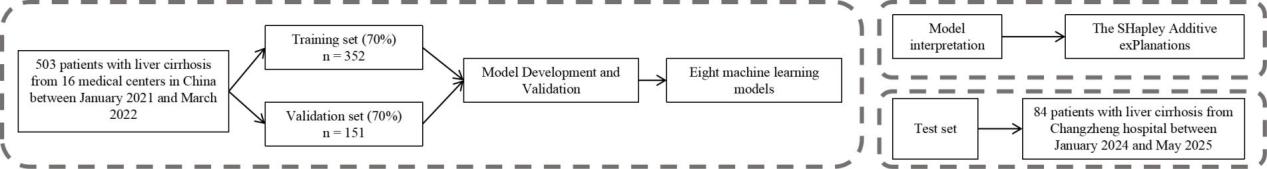


**Supplementary Figure 1.** The flowchart.

**
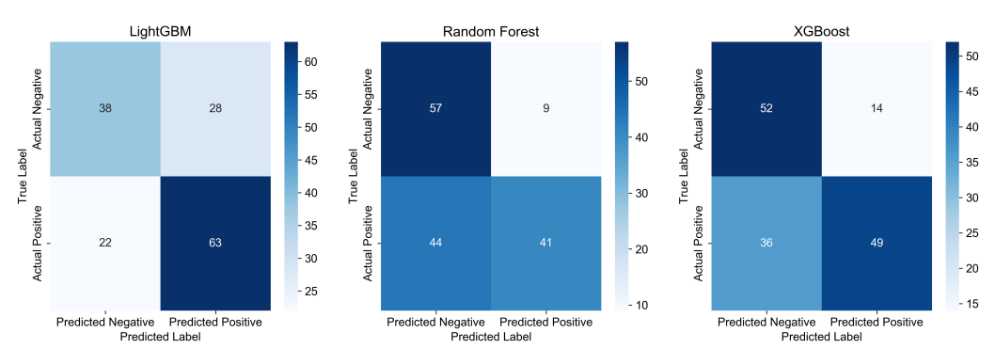
**

**Supplementary Figure 2.** The confusion matrices of the validation set.
